# Immune checkpoint inhibitor plus chemotherapy as first-line treatment for non-small cell lung cancer with malignant pleural effusion: a retrospective multicenter study

**DOI:** 10.1186/s12885-024-12173-1

**Published:** 2024-03-28

**Authors:** Qi Wei, Taibing Deng, Junhua Wu, Hao Zeng, Chang Qi, Sihan Tan, Yuanyuan Zhang, Qin Huang, Xin Pu, Weiguo Xu, Weimin Li, Panwen Tian, Yalun Li

**Affiliations:** 1 Department of Pulmonary and Critical Care Medicine, State Key Laboratory of Respiratory Health and Multimorbidity, Institute of Respiratory Health and Multimorbidity, Institute of Respiratory Health, Frontiers Science Center for Disease-related Molecular Network, Precision Medicine Center/Precision Medicine Key Laboratory of Sichuan Province, West China Hospital, Sichuan University, Chengdu, Sichuan China; 2 Lung Cancer Center/Lung Cancer Institute, West China Hospital, Sichuan University, Chengdu, Sichuan China; 3 Pulmonary and Critical Care Medicine, Guang ’an People’s Hospital, Guang ’an, China; 4 Respiratory and Critical Care Medicine, School of Medicine, Mianyang Central Hospital, University of Electronic Science and Technology of China, Mianyang, China

**Keywords:** Non-small cell lung cancer, Malignant pleural effusion, Immunotherapy, Chemotherapy, Efficacy

## Abstract

**Background:**

Immune checkpoint inhibitors (ICI) combined with chemotherapy are efficacious for treating advanced non-small cell lung cancer (NSCLC); however, the effectiveness of this approach in the malignant pleural effusion (MPE) population is unclear. This study evaluated ICI plus chemotherapy in NSCLC patients with MPE.

**Methods:**

Patients from 3 centers in China with NSCLC and MPE who received ICI plus chemotherapy (ICI Plus Chemo) or chemotherapy alone (Chemo) between December 2014 and June 2023 were enrolled. Clinical outcomes and adverse events (AEs) were compared.

**Results:**

Of 155 eligible patients, the median age was 61.0 years old. Males and never-smokers accounted for 73.5% and 39.4%, respectively. Fifty-seven and 98 patients received ICI Plus Chemo or Chemo, respectively. With a median study follow-up of 10.8 months, progression-free survival (PFS) was significantly longer with ICI Plus Chemo than with Chemo (median PFS: 7.4 versus 5.7 months; HR = 0.594 [95% CI: 0.403–0.874], *P* = 0.008). Median overall survival (OS) did not differ between groups (ICI Plus Chemo: 34.2 versus Chemo: 28.3 months; HR = 0.746 [95% CI: 0.420–1.325], *P* = 0.317). The most common grade 3 or worse AEs included decreased neutrophil count (3 [5.3%] patients in the ICI Plus Chemo group vs. 5 [5.1%] patients in the Chemo group) and decreased hemoglobin (3 [5.3%] versus 10 [10.2%]).

**Conclusions:**

In patients with untreated NSCLC with MPE, ICI plus chemotherapy resulted in significantly longer PFS than chemotherapy and had a manageable tolerability profile, but the effect on OS may be limited.

**Supplementary Information:**

The online version contains supplementary material available at 10.1186/s12885-024-12173-1.

## Introduction

Malignant pleural effusion (MPE) is a common complication of advanced tumors and significantly shortens life expectancy [1]. Approximately 125,000 hospital admissions in the USA alone are due to MPE [2]. MPE is most commonly caused by lung cancer, with non-small cell lung cancer (NSCLC) accounting for approximately one-third of case [3–5]. Patients with MPE have an average survival time of approximately 4 to 9 months, and the management of MPE is challenging in clinical practice [1, 6].

Immune checkpoint inhibitors (ICI) are an exciting new development that has dramatically altered how advanced NSCLC patients are treated in the absence of actionable oncogenic drivers. Administering ICI to patients with NSCLC and MPE is a promising treatment strategy. However, Epaillard et al. [7] conducted a study to assess clinical outcomes of patients with NSCLC and MPE were treated with ICI alone, and found that the median progression-free survival (PFS) and overall survival (OS) were just 1.8 and 6.3 months, respectively. In a retrospective multicenter study, Kawachi et al. [8] also showed that MPE was an independent predictor of reduced PFS in NSCLC patients receiving pembrolizumab alone. Thus, ICI monotherapy does not appear to be a suitable first-line treatment for NSCLC patients with MPE.

Many well-designed multi-national trials have shown that ICI plus chemotherapy substantially improves PFS and OS compared with chemotherapy alone in advanced NSCLC, irrespective of programmed cell death-ligand 1 (PD-L1) expression levels [9–11]. However, patients with pleural effusions that are uncontrolled with appropriate interventions are usually excluded from clinical trials, resulting in limited research on the efficacy and safety of the systemic combination of ICI and chemotherapy in advanced NSCLC patients with MPE [12–16]. Therefore, we conducted a retrospective study to assess the efficacy and safety of a combination therapy in patients with NSCLC and MPE.

## Methods

### Patient selection

This retrospective multicenter cohort study was conducted at 3 centers in China. We retrospectively collected medical records of NSCLC patients with MPE (stage IVA-IVB, according to the eighth edition [17]) who received a combination therapy of ICI plus chemotherapy (ICI Plus Chemo) or chemotherapy alone (Chemo) as first-line therapy between December 2014 and June 2023. The follow-up period ended on Sep 25, 2023. The inclusion criteria were as follows: (1) pathologically proven NSCLC; (2) MPE verified by histological examination of pleural tissue or cytological examination of pleural effusion; (3) no sensitizing *EGFR* mutation, *ALK* fusion or *ROS1* fusion; (4) patients receiving chemotherapy or ICI plus chemotherapy as a first line treatment; and (5) patients with comprehensive clinical data and follow-up information. Patients were excluded if they were treated with ICI alone, received less than 2 treatment cycles or were less than 18 years old. This study was approved by the West China Hospital Ethics Committee (permission number: 2022 − 1085), and informed consent was waived in accordance with the Helsinki Declaration as updated in 2013.

Data regarding age, sex, smoking status, clinical stage, metastatic sites, Eastern Cooperative Oncology Group Performance Status (ECOG PS), histological subtype, expression level of PD-L1 and treatment regimen, laboratory test results and time of commencement/progression were collected.

### End points and assessments

The primary objective was to investigate PFS and OS. Secondary outcome variables included disease control rate (DCR), objective response rate (ORR), pleurodesis success at 3 months and safety. The DCR combined rates of patients with confirmed complete response (CR), partial response (PR) and stable disease (SD). The ORR was the percentage of patients who had a confirmed CR and PR.

In the three participating research centers, patients diagnosed with advanced lung cancer are systematically followed up by their attending physicians every 8–10 weeks. During these follow-ups, a CT scan is scheduled to monitor disease progression. Radiological assessments are independently conducted by radiologists, while the attending physician evaluates the treatment efficacy based on the Response Evaluation Criteria in Solid Tumors (RECIST) version 1.1 [18]. MPE was evaluated was based on thoracic CT or ultrasound. The National Cancer Institute Common Terminology Criteria for Adverse Events (version 5.0) was used to grade the frequency, nature, and severity of adverse events (AEs) [19]. Pleurodesis was defined according to previous studies [20, 21]. If there was a lack of ipsilateral re-accumulation of MPE and the patient did not require an intervention for ipsilateral MPE during the follow-up period, pleurodesis was considered to have occurred. Recurrent and symptomatic ipsilateral MPE required pleural intervention within the follow-up period was considered pleurodesis failure. Patients without radiographic disease progression at the latest date were considered censored.

### Statistical analyses

Baseline characteristics were compared between the two groups using the Chi-Squared Test or Fisher’s exact test for categorical variables. Converting age into a dichotomous variable was performed by the age of 60 years as the cutoff point. Categorical variables are expressed as frequencies and percentages. The Kaplan-Meier method was used to analyze median PFS and median OS. Differences in proportions of pleurodesis succuss, ORR and DCR were compared by the Chi-square test. Cox proportional hazards models were used to calculate the hazard ratio (HR) and associated 95% confidence intervals (CI). The threshold for a statistically significant difference was a two-tailed *P* < 0.05. All statistical analyses were performed with R software (version 4.2.2).

## Results

### Patient characteristics

**Fig. 1:**
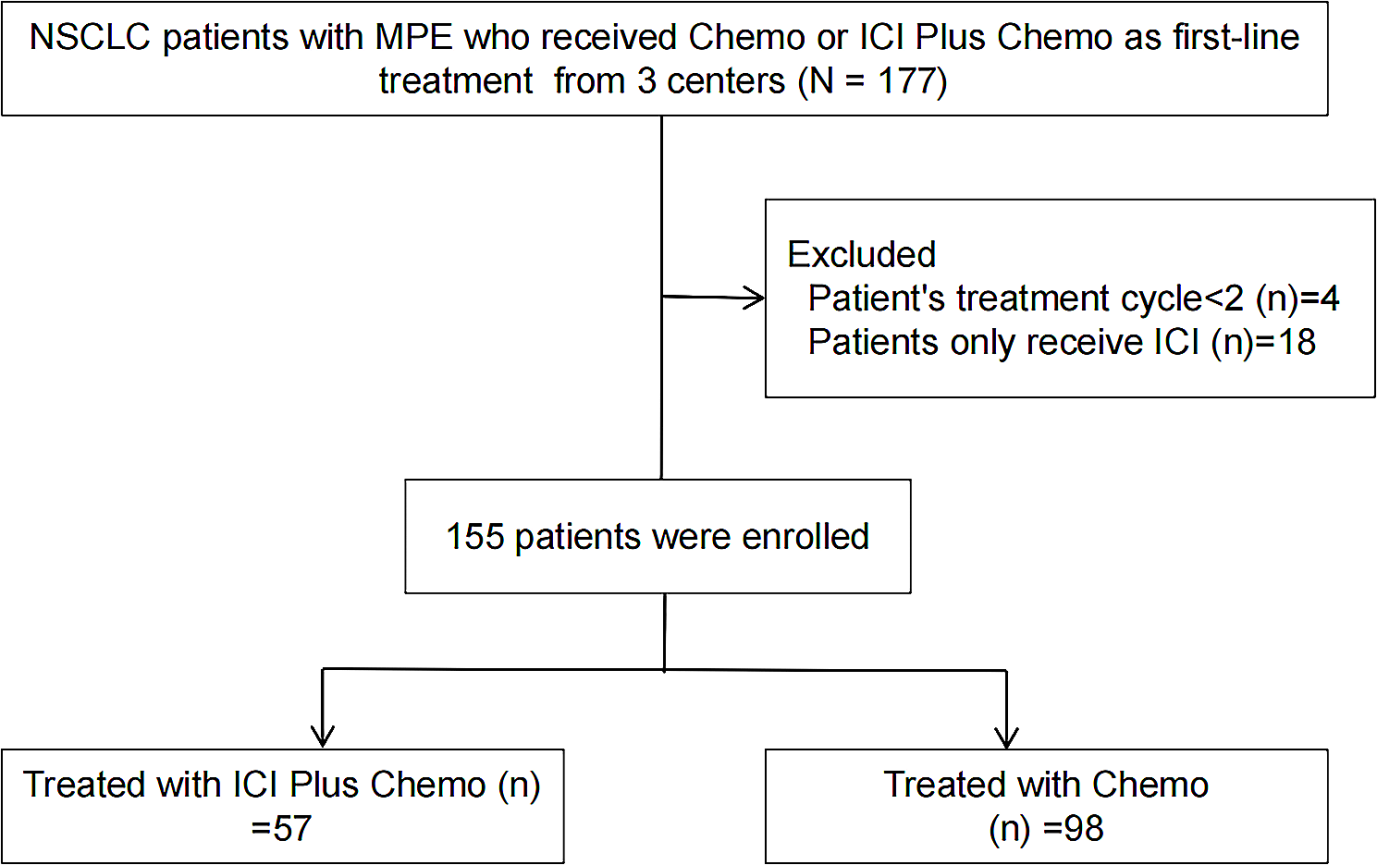
The workflow of patient selection. NSCLC, non-small cell lung cancer; MPE, malignant pleural effusion; Chemo, chemotherapy; ICI, immune checkpoint inhibitor

At the end of the data collection time period, data from 177 consecutive NSCLC patients with MPE who did not have *EGFR* mutation, *ALK* fusion or *ROS1* fusion were collected. Of these, 4 patients received less than 2 treatment cycles, and 18 patients who were treated with ICI alone were excluded. A total of 155 patients were included in our study (Fig. 1). Of the 155 patients, the median age was 61.0 years old. A total of 114 (73.5%) were male, and 41 (26.5%) were female. Current smokers, former smokers and patients who had never smoked accounted for 29.0%, 31.6 and 39.4% of the study population, respectively. A total of 113 (72.9%) patients were diagnosed with lung adenocarcinoma, 36 (23.2%) with squamous cell carcinoma and 6 (3.9%) with other cancers. During systemic therapy, 81 (52.3%) patients received intrathoracic treatment, of which 30 (19.4%) received intrathoracic administration; 74 patients (47.7%) did not. A total of 118 (76.1%) patients did not require intervention for ipsilateral MPE during the 3-month follow-up period. Fifty-seven patients received ICI Plus Chemo, and 98 patients were treated with Chemo as first-line therapy (Table 1).

**Table 1:** Patient characteristics comparison between chemotherapy group and ICI plus chemotherapy group

Patient Characteristics	Overall (*N* = 155)	Chemo (*N* = 98)	ICI Plus Chemo (*N* = 57)	*p*
Age (median [IQR])	61.00 [52.50, 68.00]	61.00 [51.00, 67.00]	64.00 [56.00, 68.00]	0.062
Age (%)				0.335
< 60y ≥ 60y	69 (44.5) 86 (55.5)	47 (48.0) 51 (52.0)	22 (38.6) 35 (61.4)	
Gender (%)				0.591
Female Male	41 (26.5) 114 (73.5)	24 (24.5) 74 (75.5)	17 (29.8) 40 (70.2)	
Smoking (%)				0.109
Current smoker	45 (29.0)	29 (29.6)	16 (28.1)	
Former smoker Never smoker	49 (31.6) 61 (39.4)	36 (36.7) 33 (33.7)	13 (22.8) 28 (49.1)	
Smoking index (%)				0.223
< 400 ≥ 400	73 (47.1) 82 (52.9)	42 (42.9) 56 (57.1)	31 (54.4) 26 (45.6)	
ECOG PS (%)				0.944
0–1 2–4	126 (81.3) 29 (18.7)	79 (80.6) 19 (19.4)	47 (82.5) 10 (17.5)	
Pathology (%)				0.864
Adenocarcinoma	113 (72.9)	70 (71.4)	43 (75.4)	
Other Squamous	6 (3.9) 36 (23.2)	4 (4.1) 24 (24.5)	2 (3.5) 12 (21.1)	
PD-L1 TPS (%)				0.026
< 1%	40 (25.8)	22 (22.4)	18 (31.6)	
≥ 50% 1–49%	8 (5.2) 21 (13.5)	4 (4.1) 9 (9.2)	4 (7.0) 12 (21.1)	
Unknown	86 (55.5)	63 (64.3)	23 (40.4)	
Stage (%)				0.564
IVA IVB	109 (70.3) 46 (29.7)	71 (72.4) 27 (27.6)	38 (66.7) 19 (33.3)	
Brain metastasis (%)				0.45
No Yes	136 (87.7) 19 (12.3)	84 (85.7) 14 (14.3)	52 (91.2) 5 (8.8)	
Bone metastasis (%)				0.764
No Yes	115 (74.2) 40 (25.8)	74 (75.5) 24 (24.5)	41 (71.9) 16 (28.1)	
Liver metastasis (%)				1
No Yes	145 (93.5) 10 (6.5)	92 (93.9) 6 (6.1)	53 (93.0) 4 (7.0)	
Metastasis				0.86
Multiple organ metastasis	103 (66.5)	64 (65.3)	39 (68.4)	
Single organ metastasis	52 (33.5)	34 (34.7)	18 (31.6)	
NLR				0.968
< 5 ≥ 5	105 (67.7) 50 (32.3)	67 (68.4) 31 (31.6)	38 (66.7) 19 (33.3)	
Bevacizumab (%) No Yes	137 (88.4) 18 (11.6)	80 (81.6) 18 (18.4)	57 (100.0) 0 (0.0)	0.001
Intrathoracic treatment (%)				0.812
No Yes	74 (47.7) 81 (52.3)	48 (49.0) 50 (51.0)	26 (45.6) 31 (54.4)	
Intrathoracic administration (%)				0.518
No Yes	125 (80.6) 30 (19.4)	77 (78.6) 21 (21.4)	48 (84.2) 9 (15.8)	
Pleurodesis success at 3 months (%)				0.983
Fail	32 (20.6)	20 (20.4)	12 (21.1)	
Success	118 (76.1)	75 (76.5)	43 (75.4)	
Unknown	5 (3.2)	3 (3.1)	2 (3.5)	

*Abbreviations*: *ICI* Immune checkpoint inhibitor, *Chemo* Chemotherapy, *IQR* interquartile range, *ECOG PS* Eastern Cooperative Oncology Group Performance Status, *PD-L1 TPS* Programmed cell death-Ligand 1 Tumor cell Proportion Score, *NLR* Neutrophil to Lymphocyte ratio

Baseline characteristics were generally well balanced between the groups, except for the proportion of patients with a PD-L1 tumor proportion score of 1% or higher (16 [28.1%] of 57 patients in the ICI Plus Chemo group vs. 13 [13.3%] of 98 patients in the Chemo group) and the percentage of patients who were administered bevacizumab intravenously (0 of 57 patients in the ICI Plus Chemo group vs. 18 [18.4%] of 98 patients in the Chemo group) (Table 1). Among these patients, there were missing values for PD-L1 tumor proportion score (TPS); the Fisher test was performed after excluding the missing values for each item, and no significant difference between the two groups for PD-L1 TPS was observed.

### Efficacy and response assessment

The ORR was 42.6% in the ICI Plus Chemo group and 35.2% in the Chemo group (*P* = 0.484) (Fig. S1A). The DCRs were 85.2% and 81.8%, respectively (*P* = 0.773) (Fig. S1B). A similar rate of pleurodesis success at 3 months was observed in ICI Plus Chemo (78.2%) compared with Chemo alone (78.9%) (*P* = 1.000) (Fig. S1C).

**Fig. 2:**
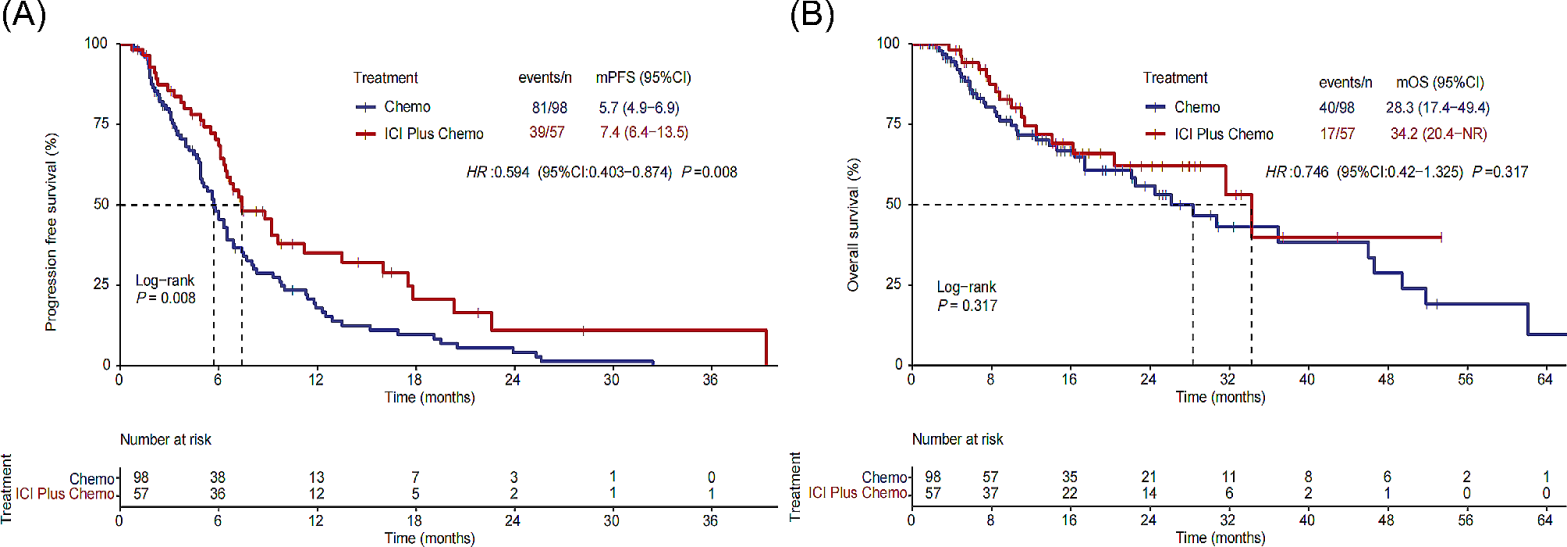
Kaplan-Meier curve of PFS (2 **A**) and OS (2**B**) in Chemo group and ICI Plus Chemo group. PFS, progression-free survival, OS, overall survival; Chemo, chemotherapy; ICI, immune checkpoint inhibitor

**Fig. 3:**
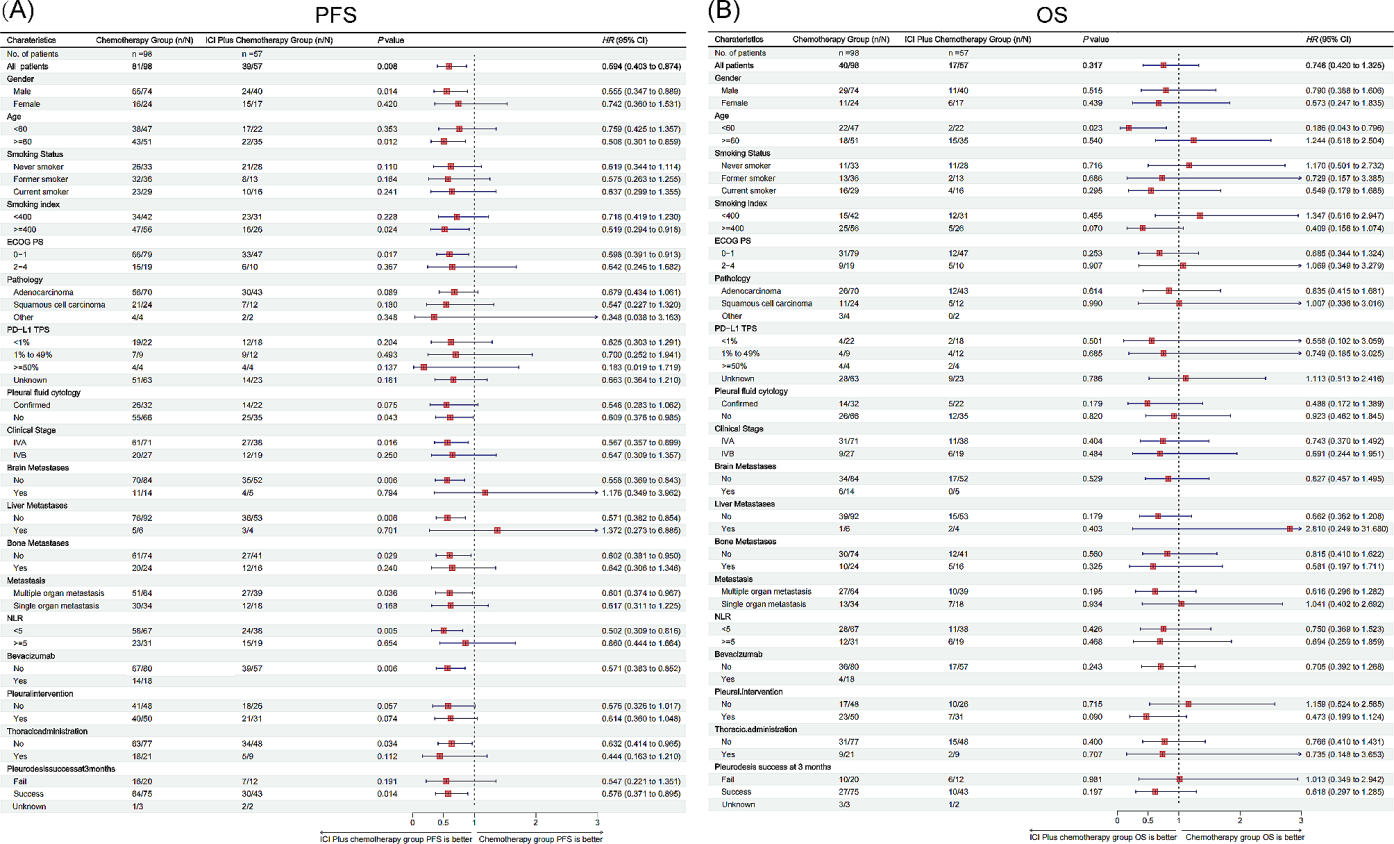
Subgroup analysis of PFS (3 **A**) and OS (3**B**) in patients received chemotherapy and ICI plus chemotherapy. HR: hazard ratio; CI, confidence interval; ECOG PS: Eastern Cooperative Oncology Group Performance Status; PD-L1, programmed cell death-ligand 1; NLR: Neutrophil to Lymphocyte ratio; ICI, immune checkpoint inhibitor; PFS, progression-free survival

With a median study follow-up of 10.8 (5.7, 22.2) months, PFS was significantly longer with ICI Plus Chemo than with Chemo (median PFS: 7.4 versus 5.7 months; HR = 0.594 [95% CI: 0.403–0.874], *P* = 0.008) (Fig. 2A). In most subgroups evaluated, the observed PFS benefit was maintained with ICI Plus Chemo versus Chemo (Fig. 3A). The median OS did not differ between the ICI Plus Chemo and Chemo groups (median OS: 34.2 versus 28.3 months; HR = 0.746 [95% CI: 0.420–1.325], *P* = 0.317) (Fig. 2B). Additionally, the results of the subgroup analyses were also consistent, except for patients younger than 60, in whom ICI Plus Chemo showed improved OS versus Chemo (Fig. 3B). In addition, in patients with a PD-L1 TPS of less than 1%, the HRs for PFS and OS were 0.625 (95% CI: 0.303–1.291) and 0.558 (95% CI 0.102–3.059), respectively, for patients treated with ICI Plus Chemo versus Chemo. In patients with PD-L1 TPS expression levels between 1% and 49%, the HRs for PFS and OS were 0.700 (95% CI: 0.252–1.941) and 0.749 (95% CI: 0.185–3.025), respectively.

Furthermore, to explore the effect of intravenous infusion of bevacizumab on PFS and OS, we divided patients in the Chemo group into a chemotherapy group and a chemotherapy plus bevacizumab group. PFS was also significantly longer with ICI Plus Chemo compared with chemotherapy alone and chemotherapy plus bevacizumab (median PFS: 7.4 versus 5.6 months versus 6.5 months, *P* = 0.019) (Fig. S2A). The median OS was not significantly different among the ICI Plus Chemo, chemotherapy and chemotherapy plus bevacizumab groups (median OS: 34.2 months, 28.3 months and 26.1 months, respectively, *P* = 0.460) (Figure S2B).

### Safety assessment

**Fig. 4:**
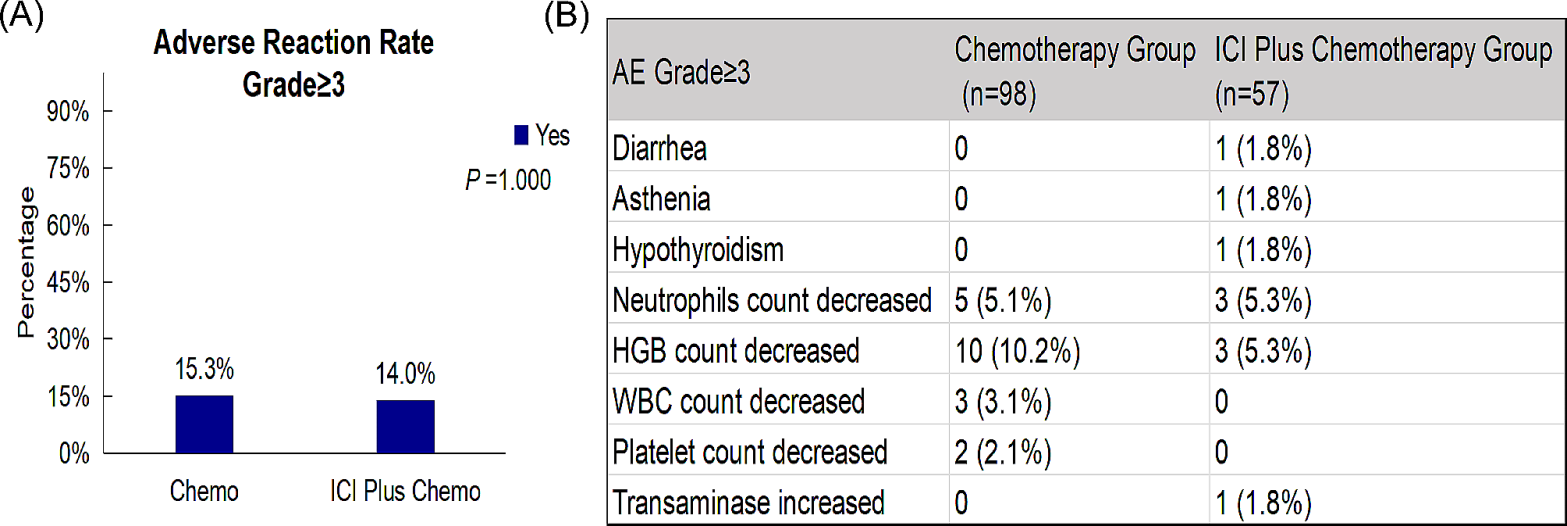
The histogram of treatment-related adverse events rate of all grade and grade ≥ 3 in Chemo group and ICI Plus Chemo group

There was no obvious distinction between the two groups in grade ≥ 3 AEs, with 14.0% (8/57) of patients in the ICI Plus Chemo group and 15.3% (15/97) of patients in the Chemo group experiencing at least one (*P* = 1.000) (Fig. 4A).

The most common types of the grade ≥ 3 AEs included decreased neutrophil count (3 [5.3%] patients in the ICI Plus Chemo group vs. 5 [5.1%] patients in the Chemo group) and decreased hemoglobin (3 [5.3%] versus 10 [10.2%]). The grade ≥ 3 AEs that had a difference of 2% or more between the ICI Plus Chemo group and Chemo group was decreased hemoglobin (Fig. 4B).

## Discussion

To our knowledge, this is the first study to examine the efficacy and safety of ICI plus chemotherapy compared with chemotherapy as a first-line treatment for NSCLC patients with MPE. In this retrospective multicenter cohort study, we found that ICI plus chemotherapy resulted in a significant, clinically meaningful improvement in PFS in patients with advanced NSCLC with MPE. However, the effect of the combined therapy on OS was limited.

It is well known that MPE is associated with high levels of IL-6, CCL2, VEGF, TGF-β, and HIF, all of which are associated with maintaining a tumor phenotype resembling stem cells [22, 23]. According to Bruschini and colleagues, the total number of effector cells in MPE, including T lymphocytes and NK cells, declines [24]. On the other hand, a large percentage of M2 polarized macrophages are discovered in MPE, which are well-known to engage in proangiogenic and metastatic pathways. The above studies show that the MPE of NSCLC is an immunosuppressive and tumor-promoting environment. Nevertheless, our study demonstrated that ICI plus chemotherapy prolonged the PFS of patients with NSCLC and MPE compared with Chemo.

There are many possible reasons to explain the findings of this study. First, a previous study discovered that PD-L1 expression was highly consistent across histological specimens and matched pleural fluid from NSCLC patients, implying that if the primary tumor is responsive to anti-PD-L1 treatment, MPE may also respond [25]. Second, Li and colleagues conducted a study of intrathoracic injections of anti-PD1 monoclonal antibody (mAb) to manage MPE, and it is worth noting that caudal intravenous and intrathoracic injections of anti-PD1 mAb yielded similar results in MPE control [4]. These results suggested that systemic anticancer agents such as ICI may be efficacious in MPE patients. Additionally, chemotherapy can enhance the efficacy of ICIs. Several preclinical studies have suggested that chemotherapy may alter the tumor microenvironment [26]. Chemotherapy can trigger the release of the chemokine CXCL10 and the rapid secretion of type I IFNs, which can recruit CD8 + and CD4 + effector T cells to tumor sites and boost antitumor immunity [27]. Research has demonstrated that chemotherapy can disrupt the activity of regulatory T cells (Tregs) and improve early dendritic cell maturation and function via TLR4 signaling [28, 29]. Furthermore, chemotherapy may boost the immune response by inducing apoptosis in tumor cells and increasing the expression of MHC class I molecules of the cGAS-STING pathway, which is essential in this process [30, 31]. Previous investigations have shown that chemotherapy can stimulate the tumor immune microenvironment, resulting in a synergistic enhancement of ICI.

Notably, our study revealed that in patients with NSCLC and MPE, the role of ICI plus Chemo for OS may be limited compared with chemo. Similarly, Kawachi et al. conducted a multicenter retrospective study to evaluate the efficacy of ICIs with or without chemotherapy for patients with NSCLC and MPE [32]. They demonstrated that the group receiving ICI and chemotherapy showed a similar median OS compared with patients receiving ICI monotherapy (22.7 months versus 19.9 months, *P* = 0.071). While overall survival (OS) is indeed considered the definitive criterion for evaluating treatment efficacy, practical challenges often preclude its use as the sole endpoint in clinical studies [33–35]. This discrepancy can be attributed to several factors. Firstly, the impact of subsequent lines of therapy can dilute OS results, complicating the achievement of statistically significant differences without negating the therapeutic benefit. Secondly, non-cancer-related mortality can also influence OS outcomes. Lastly, the requirement for a large sample size to demonstrate a difference using OS as a primary endpoint poses its own set of challenges. In our study, the limited number of events within each treatment group suggests that the follow-up duration may have been insufficient to fully assess long-term survival benefits. Future studies with larger cohorts are anticipated to provide more definitive insights. It is well known that MPE has historically been associated with a grim prognosis. Previous studies have shown that the presence of MPE is related to reduced ICI efficacy, notably reduced OS [36, 37]. In the era of immunotherapy and antiangiogenic therapy, future studies should focus on establishing a series of intrapleural therapies and systemic therapies to improve the OS of MPE patients.

Our study’s safety data are detailed in Fig. 4, where we noted a lower incidence of treatment-related adverse events (TRAEs) Grade ≥ 3 compared to recent clinical trials, as evidenced by Camrelizumab combined with chemotherapy showing a 29.3% incidence (60 patients) and chemotherapy alone showing an 11.1% incidence (23 patients) [38]. In our study, the incidence was 14.0% (8/57) in the ICI Plus Chemo group and 15.3% (15/97) in the Chemo group. Given the retrospective nature of our study, it is acknowledged that adverse events may not be captured with the same granularity as in prospective clinical trials. This inherent limitation may contribute to the observed discrepancy in the rate of TRAEs. Nonetheless, the adverse events recorded in our study align with those reported in clinical trials, suggesting that the safety profile of ICI plus chemotherapy in advanced NSCLC with MPE remains manageable.

The are several limitations to our study. First, this study included patients only in China, which precludes the generalizability of the results to patients in other countries. Second, the effectiveness of ICI plus chemotherapy in treating MPE is still subject to clinical trials, as this was a retrospective study with a somewhat limited sample size. And then the rate of adverse reactions in this study was lower than in clinical trials as this was a retrospective study that did not adequately identify adverse reactions. Despite these findings, our study maintained sufficient statistical power to show a significant PFS difference between the ICI Plus Chemo group and Chemo group. Finally, no patients received antiangiogenic therapy in the ICI Plus Chemo group. Therefore, we could not explore whether ICI plus chemotherapy and antiangiogenic therapy improves the OS of patients with MPE.

## Conclusion

In untreated NSCLC patients with MPE, ICI plus chemotherapy resulted in significantly longer PFS than chemotherapy alone and had a manageable tolerability profile. However, the effect of the combined therapy on OS may be limited. Randomized clinical trials using ICI plus chemotherapy for patients with NSCLC and MPE are needed to further elucidate the clinical effect of our findings.

### Electronic supplementary material

Below is the link to the electronic supplementary material.

Supplementary Material 1

### Electronic supplementary material

Below is the link to the electronic supplementary material.

Supplementary Material 2

## Data Availability

No datasets were generated or analysed during the current study.

## Abbreviations

MPE: Malignant pleural effusion
NSCLC: Non-small cell lung cancer
ICI: Immune checkpoint inhibitors
PFS: Progression-free survival
OS: Overall survival
PD-L1: Programmed cell death-ligand 1
ECOG: PS Eastern Cooperative Oncology Group Performance Status
DCR: Disease control rate
ORR: Objective response rate
CR: Complete response
PR: Partial response
SD: Stable disease
CT: Computed tomography
MRI: Magnetic resonance imaging
RECIST: Response Evaluation Criteria in Solid Tumor
HR: Hazard ratio
CI: Confidence intervals

## References

[1] Zamboni MM, da Silva CT, Baretta R, Cunha ET, Cardoso GP (2015). Important prognostic factors for survival in patients with malignant pleural effusion. BMC Pulm Med.

[2] Taghizadeh N, Fortin M, Tremblay A (2017). US hospitalizations for malignant pleural effusions: data from the 2012 National Inpatient Sample. Chest.

[3] Froudarakis ME (2012). Pleural effusion in lung cancer: more questions than answers. Respiration.

[4] Li X, Wu G, Chen C, Zhao Y, Zhu S, Song X (2021). Intrapleural injection of Anti-PD1 antibody: a Novel Management of Malignant Pleural Effusion. Front Immunol.

[5] Penz E, Watt KN, Hergott CA, Rahman NM, Psallidas I (2017). Management of malignant pleural effusion: challenges and solutions. Cancer Manag Res.

[6] Morgensztern D, Waqar S, Subramanian J, Trinkaus K, Govindan R (2012). Prognostic impact of malignant pleural effusion at presentation in patients with metastatic non-small-cell lung cancer. J Thorac Oncol.

[7] Epaillard N, Benitez JC, Gorria T, Fabre E, Riudavets M, Reyes R (2021). Pleural effusion is a negative prognostic factor for immunotherapy in patients with non-small cell lung cancer (NSCLC): the pluie study. Lung Cancer.

[8] Kawachi H, Tamiya M, Tamiya A, Ishii S, Hirano K, Matsumoto H (2020). Association between metastatic sites and first-line pembrolizumab treatment outcome for advanced non-small cell lung cancer with high PD-L1 expression: a retrospective multicenter cohort study. Invest New Drugs.

[9] Paz-Ares L, Luft A, Vicente D, Tafreshi A, Gümüş M, Mazières J (2018). Pembrolizumab plus Chemotherapy for squamous non-small-cell Lung Cancer. N Engl J Med.

[10] Awad MM, Gadgeel SM, Borghaei H, Patnaik A, Yang JCH, Powell SF (2021). Long-term overall survival from KEYNOTE-021 cohort G: Pemetrexed and Carboplatin with or without Pembrolizumab as First-Line Therapy for Advanced Nonsquamous NSCLC. J Thorac Oncol.

[11] Reck M, Rodríguez-Abreu D, Robinson AG, Hui R, Csőszi T, Fülöp A (2019). Updated analysis of KEYNOTE-024: Pembrolizumab Versus Platinum-based chemotherapy for Advanced Non-small-cell Lung Cancer with PD-L1 Tumor Proportion score of 50% or Greater. J Clin Oncol.

[12] Zhou C, Chen G, Huang Y, Zhou J, Lin L, Feng J (2021). Camrelizumab plus carboplatin and pemetrexed versus chemotherapy alone in chemotherapy-naive patients with advanced non-squamous non-small-cell lung cancer (CameL): a randomised, open-label, multicentre, phase 3 trial. Lancet Respir Med.

[13] Zhou C, Wang Z, Sun Y, Cao L, Ma Z, Wu R (2022). Sugemalimab versus placebo, in combination with platinum-based chemotherapy, as first-line treatment of metastatic non-small-cell lung cancer (GEMSTONE-302): interim and final analyses of a double-blind, randomised, phase 3 clinical trial. Lancet Oncol.

[14] Zhou C, Wu L, Fan Y, Wang Z, Liu L, Chen G (2021). Sintilimab Plus Platinum and Gemcitabine as First-Line treatment for Advanced or metastatic squamous NSCLC: results from a Randomized, Double-Blind, phase 3 trial (ORIENT-12). J Thorac Oncol.

[15] Shiraishi Y, Kishimoto J, Sugawara S, Mizutani H, Daga H, Azuma K et al. Atezolizumab and Platinum Plus Pemetrexed with or without Bevacizumab for Metastatic Nonsquamous Non-small Cell Lung Cancer: a phase 3 Randomized Clinical Trial. JAMA Oncol. 2023;e235258. 10.1001/jamaoncol.2023.5258 PMC10739077 38127362

[16] Wang Z, Wu L, Li B, Cheng Y, Li X, Wang X (2023). Toripalimab Plus Chemotherapy for patients with treatment-naive Advanced Non-small-cell Lung Cancer: a Multicenter Randomized Phase III Trial (CHOICE-01). J Clin Oncol.

[17] Goldstraw P, Chansky K, Crowley J, Rami-Porta R, Asamura H, Eberhardt WEE (2016). The IASLC Lung Cancer Staging Project: proposals for revision of the TNM Stage groupings in the Forthcoming (Eighth) Edition of the TNM classification for Lung Cancer. J Thorac Oncol.

[18] Eisenhauer EA, Therasse P, Bogaerts J, Schwartz LH, Sargent D, Ford R (2009). New response evaluation criteria in solid tumours: revised RECIST guideline (version 1.1). Eur J Cancer.

[19] Freites-Martinez A, Santana N, Arias-Santiago S, Viera A (2021). Using the common terminology criteria for adverse events (CTCAE - version 5.0) to evaluate the severity of adverse events of Anticancer therapies. Actas Dermosifiliogr (Engl Ed).

[20] Chaddha U, Agrawal A, Bhavani SV, Sivertsen K, Donington DJ, Ferguson MK (2021). Thoracic ultrasound as a predictor of pleurodesis success at the time of indwelling pleural catheter removal. Respirology.

[21] Suzuki K, Servais EL, Rizk NP, Solomon SB, Sima CS, Park BJ (2011). Palliation and pleurodesis in malignant pleural effusion: the role for tunneled pleural catheters. J Thorac Oncol.

[22] Hayama N, Hattori S, Takahashi G, Takahashi F, Takeuchi T, Tanaka J (2020). Cytokine/Chemokine/Growth factor levels in Malignant Pleural Effusion of Non-small Cell Lung Cancer. Tokai J Exp Clin Med.

[23] Donnenberg AD, Luketich JD, Dhupar R, Donnenberg VS (2019). Treatment of malignant pleural effusions: the case for localized immunotherapy. J Immunother Cancer.

[24] Bruschini S, Pallocca M, Sperandio E, D’Ambrosio L, Ascenzi F, De Vitis C (2022). Deconvolution of malignant pleural effusions immune landscape unravels a novel macrophage signature associated with worse clinical outcome in lung adenocarcinoma patients. J Immunother Cancer.

[25] Grosu HB, Arriola A, Stewart J, Ma J, Bassett R, Hernandez M (2019). PD-L1 detection in histology specimens and matched pleural fluid cell blocks of patients with NSCLC. Respirology.

[26] Emens LA, Middleton G (2015). The interplay of immunotherapy and chemotherapy: harnessing potential synergies. Cancer Immunol Res.

[27] Sistigu A, Yamazaki T, Vacchelli E, Chaba K, Enot DP, Adam J (2014). Cancer cell-autonomous contribution of type I interferon signaling to the efficacy of chemotherapy. Nat Med.

[28] Ercolini AM, Ladle BH, Manning EA, Pfannenstiel LW, Armstrong TD, Machiels JPH (2005). Recruitment of latent pools of high-avidity CD8(+) T cells to the antitumor immune response. J Exp Med.

[29] Pfannenstiel LW, Lam SSK, Emens LA, Jaffee EM, Armstrong TD (2010). Paclitaxel enhances early dendritic cell maturation and function through TLR4 signaling in mice. Cell Immunol.

[30] Li T, Chen ZJ (2018). The cGAS-cGAMP-STING pathway connects DNA damage to inflammation, senescence, and cancer. J Exp Med.

[31] Pépin G, Gantier MP (2017). cGAS-STING activation in the Tumor Microenvironment and its role in Cancer Immunity. Adv Exp Med Biol.

[32] Kawachi H, Tamiya M, Taniguchi Y, Yokoyama T, Yokoe S, Oya Y (2022). Efficacy of Immune checkpoint inhibitor with or without chemotherapy for Nonsquamous NSCLC with Malignant Pleural Effusion: a retrospective Multicenter Cohort Study. JTO Clin Res Rep.

[33] Gill S, Berry S, Biagi J, Butts C, Buyse M, Chen E (2011). Progression-free survival as a primary endpoint in clinical trials of metastatic colorectal cancer. Curr Oncol.

[34] Lu S, Wang J, Yu Y, Yu X, Hu Y, Ai X (2021). Tislelizumab Plus Chemotherapy as First-Line treatment for locally advanced or metastatic nonsquamous NSCLC (RATIONALE 304): a randomized phase 3 trial. J Thorac Oncol.

[35] Merino M, Kasamon Y, Theoret M, Pazdur R, Kluetz P, Gormley N (2023). Irreconcilable differences: the Divorce between Response Rates, Progression-Free Survival, and overall survival. J Clin Oncol.

[36] Shibaki R, Murakami S, Shinno Y, Matsumoto Y, Goto Y, Kanda S (2019). Malignant pleural effusion as a predictor of the efficacy of anti-PD-1 antibody in patients with non-small cell lung cancer. Thorac Cancer.

[37] Wong T, Fuld AD, Feller-Kopman DJ (2023). Malignant pleural effusions in the era of Immunotherapy and Antiangiogenic Therapy. Semin Respir Crit Care Med.

[38] Zhou C, Chen G, Huang Y, Zhou J, Lin L, Feng J (2023). Camrelizumab Plus Carboplatin and Pemetrexed as First-Line treatment for Advanced Nonsquamous NSCLC: Extended Follow-Up of CameL phase 3 trial. J Thorac Oncol.

