# Supplementary figures and images for: Immune checkpoint inhibitor plus chemotherapy as first-line treatment for non-small cell lung cancer with malignant pleural effusion: a retrospective multicenter study
Source: BMC Cancer. 2024 Mar 28;24:393. doi: 10.1186/s12885-024-12173-1 (PMC10976680; doi:10.1186/s12885-024-12173-1)

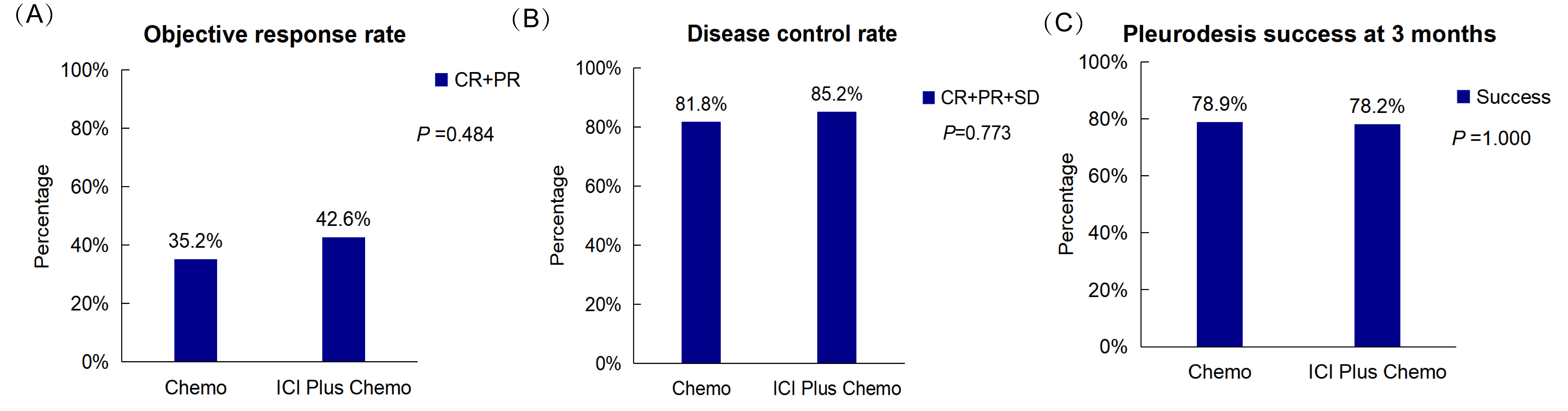

Supplement: Supplementary file 1 — Supplementary Material 1 [file 12885_2024_12173_MOESM1_ESM.png]

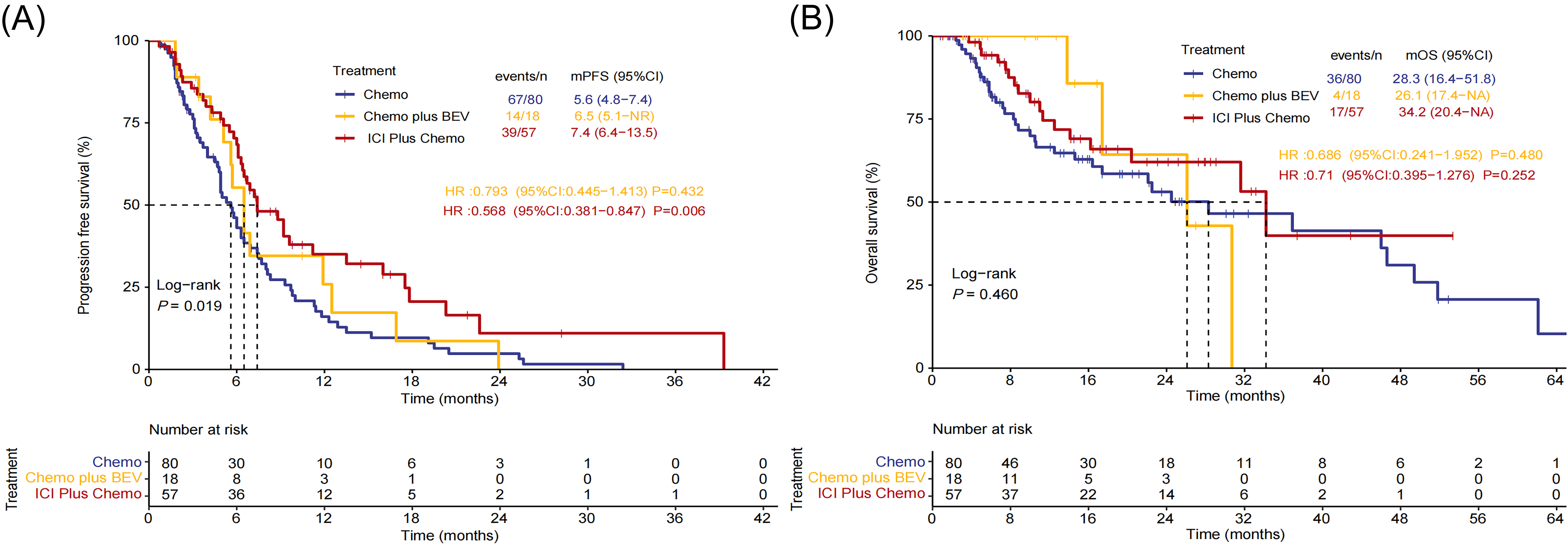

Supplement: Supplementary file 2 — Supplementary Material 2 [file 12885_2024_12173_MOESM2_ESM.png]
